# Validation of a Spanish Version of the Lille Apathy Rating Scale for Parkinson's Disease

**DOI:** 10.1155/2014/849834

**Published:** 2014-10-14

**Authors:** Rocio García-Ramos, Clara Villanueva Iza, María José Catalán, Abilio Reig-Ferrer, Jorge Matías-Guíu

**Affiliations:** ^1^Neurology Service, Hospital Clinico San Carlos, IdiSSC, Universidad Complutense, Calle Profesor, Martin Lagos s/n, 28040 Madrid, Spain; ^2^Facultad de Ciencias de la Salud, Universidad de Alicante, 03080 Alicante, Spain

## Abstract

*Introduction*. To date, no rating scales for detecting apathy in Parkinson's disease (PD) patients have been validated in Spanish. For this reason, the aim of this study was to validate a Spanish version of Lille apathy rating scale (LARS) in a cohort of PD patients from Spain. *Participants and Methods*. 130 PD patients and 70 healthy controls were recruited to participate in the study. Apathy was measured using the Spanish version of LARS and the neuropsychiatric inventory (NPI). Reliability (internal consistency, test-retest, and interrater reliability) and validity (construct, content, and criterion validity) were measured. *Results*. Interrater reliability was 0.93. Cronbach's *α* for LARS was 0.81. The test-retest correlation coefficient was 0.97. The correlation between LARS and NPI scores was 0.61. The optimal cutoff point under the ROC curve was −14, whereas the value derived from healthy controls was −11. The prevalence of apathy in our population tested by LARS was 42%. *Conclusions*. The Spanish version of LARS is a reliable and useful tool for diagnosing apathy in PD patients. Total LARS score is influenced by the presence of depression and cognitive impairment. However, both disorders are independent identities with respect to apathy. The satisfactory reliability and validity of the scale make it an appropriate instrument for screening and diagnosing apathy in clinical practice or for research purposes.

## 1. Introduction

Between 16 and 48% of Parkinson's disease (PD) patients develop apathy during the course of the disease [1, 2]. Apathy supposes a huge impact on the quality of life of the PD patients and their families [3]. Apathetic patients have a very important reduction in the activities of daily living appropriate to their age, independent of other aspects of the disease. Hence diagnosing this syndrome is clue to evaluate these patients. Apathy's rating scales are useful tools for this and they are important to evaluate future therapeutic interventions.

A series of rating scales have been proposed to identify and quantify apathy and to differentiate it from other disorders, especially depression [4]. In 1991 Marin et al. [5] proposed the apathy evaluation scale (AES) based on his conceptual definition of apathy: “lack of motivation not attributable to diminished level of consciousness, cognitive impairment, or emotional distress” [6]. Starkstein et al. [7] adapted and extended Marin's definition to establish a series of standardized diagnostic criteria which constituted the apathy scale (AS). In 1994, Cummings et al. [8] developed a tool for assessing behavioural disturbances in patients with dementia, called the Neuropsychiatric Inventory (NPI); it included one subscale focusing on apathy. In 2002 Robert et al. extended NPI by specifically measuring “emotional blunting, lack of initiative, and lack of interest.” This rating scale was named the apathy inventory (AI) [9]. In addition to these available tools, item 4 of the Unified Parkinson's Disease Rating Scale (UPDRS) has also been used for detecting apathy [10].

In 2006, Sockeel et al. [11] proposed the Lille apathy rating scale (LARS) as a tool to detect and quantify apathy and distinguish it from depression in PD patients. Two years later, these authors validated a caregiver-based version of the scale [12]. LARS was created based on Marin's AES criteria but also extended taking into account the pathophysiological processes underlying apathy [13]. The psychometric properties of LARS make it appropriate for measuring the disorder in these patients. For this reason, the aim of this study was to validate a Spanish version of LARS in a cohort of PD patients from Spain.

## 2. Participants and Methods

Two hundred participants were recruited from the Movement Disorder Unit at Hospital Clínico San Carlos (Madrid, Spain). The total included 130 individuals in the PD patient group and 70 healthy controls. PD was diagnosed according to the United Kingdom Parkinson's Disease Society Brain Bank Criteria [14]. Consecutive PD treated patients were recruited in three months. The control group consisted of caregivers or patients' companions who were not diagnosed with PD. The inclusion criteria to participate in the study were as follows: age over 18; no acute systemic diseases or central nervous system disorders such as Alzheimer disease, cerebrovascular disease or epilepsy; good health during at least the last three months before recruitment; and no visual, hearing, or physical impairments. Furthermore, specifically for the control group, subjects had to score higher than 26 on the 35-point Mini-Mental Status Examination (MMSE) and less than 13 on Montgomery-Åsberg depression rating scale (MADRS).

Demographic and clinical information from participants was collected at the time of recruitment and the clinical diagnostic of apathy was made at this moment for the main researcher based on the Starkstein and Leentjens's criteria of apathy [15]. All patients and control group gave their informed consent to participate in the study. Procedures were performed in accordance with guidelines established by the Ethics Committee at Hospital and the study was accepted for this committee.

### 2.1. Lille Apathy Rating Scale

The characteristics of LARS have been explained elsewhere [11]. Briefly, the scale is composed of 33 items grouped in 9 domains, or subscales, which evaluate “reduction in everyday productivity (EP), lack of interest (INT), lack of initiative (INI), extinction of novelty seeking (NS) and motivation (M), blunting of emotional responses (ER), lack of concern (C), poor social life (SL), and extinction of self-awareness (SA)” [11]. Each item was worded as a simple, clear question to which the participant responded with a yes or no. The items refer to emotions and activities performed during the four weeks prior to the interview. The total LARS score ranges from −36 to +36; higher positive scores indicate increased degrees of apathy. Dr. Sockeel gave consent for the preparation of a Spanish version of LARS. Although published in English [11], the original language of LARS is French. The standard “forward-backward” procedure was applied to translate the LARS. Two bilinguals translated the original scale into Spanish.

Two independent translators then back-translated the two translated version into English. The translators were not connected to the study, so comparability and meaning equivalence were ensured. Using the different versions, the authors created a provisional Spanish version of the LARS. An independent professional revised this version. Minor differences were corrected at this stage by agreement between the different translations and the final version was made available.

### 2.2. Experimental Phases and Additional Scales

In order to validate LARS, two studies were performed. (1) A pilot study assessing participants' item comprehension, the format and applicability of the scale, and the interrater reliability of the proposed Spanish version of LARS. With that aim, a total of 30 PD patients were interviewed individually by two investigators after being evaluated using the rating scale. Interrater reliability was measured by calculating the Kappa coefficient (*κ*) [16]. (2) An experimental study validates the psychometric properties of the scale and assesses apathy among subjects. Motor disability and progression of disease were evaluated in PD patients by means of the UPDRS and Hoehn and Yahr scale, respectively [17–19]. In addition to LARS, NPI apathy subscale was used to measure apathy in PD patients. Controls were evaluated with LARS scale too. Moreover, all 200 participants (patients and healthy controls) were assessed for a blinded neuropsychologist for depression, dementia, and cognitive impairment using MADRS, Mattis Dementia Rating Scale (MATTIS), Clock Drawing Test, the assessment of semantic and phonemic fluency, and MEC [20–22]. In order to determine the test-retest reliability of LARS, 30 of the PD patients repeated the interview 15 days after the initial session.

### 2.3. Data and Statistical Analysis

Since tests were administered by the researcher, 100% of data were fully computable. The main psychometrics properties, that is, reliability (determined by internal consistency and test-retest reliability, interrater reliability) and validity (construct, content, and criterion validity), were measured for the Spanish version of LARS. Internal consistency was determined by calculating the correlation matrix of the 33 items on the scale (using Pearson's coefficient), Cronbach's alpha coefficient (*α*), and split-half reliability (using the Spearman-Brown formula). Test-retest reliability was calculated using the intraclass correction coefficient (ICC) and paired Student's *t*-test. Construct validity was evaluated by factor extraction using principal component analysis (PCA). Oblique rotation was performed to interpret factor loadings. Convergent validity was measured by analysing Pearson's correlation between LARS and NPI scores. The diagnostic accuracy of LARS was determined by comparing the total LARS score and the clinical diagnosis established by the main researcher based in Starkstein and Leentjens's criteria, using a receiver operating characteristic (ROC) curve and calculating the area under the curve (AUC).

Sensitivity, specificity, accuracy, and the Kappa coefficient were measured at those cutoff points on the ROC curve with an optimal sensitivity and specificity pair. Floor and ceiling effects were identified when an item received the minimum or the maximum scores, respectively, from more than 90% of participants. The item discrimination index was evaluated by comparing the score on each item to the total LARS score, including one-third of both the highest and the lowest scores. The relationship between apathy (diagnosed according to the main researcher's clinical criteria) and depression (determined by MADRS) or cognitive impairment (by MATTIS) was measured by two-factor analysis of variance (ANOVA).

The correlation between the total LARS score and demographic and clinical characteristics of subjects was analysed using either Pearson (parametric) or Spearman (nonparametric) correlation coefficients. Multiple regression analysis was performed to identify characteristics independently related to the total LARS score; variables presenting a *P* ≤ 0.05 or a correlation coefficient ≥0.20 were included in the univariate analysis. Demographic and clinical characteristics were compared among PD patients and healthy controls using Student's *t*-test for continuous variables and the chi-square test or Fisher's exact test for categorical ones. The latter were expressed as frequencies, whereas continuous variables were expressed as mean ± standard deviation (SD). Statistical significance was considered when *P* ≤ 0.05. All the statistical procedures were performed using SPSS 15.0 (IBM, Chicago, USA).

## 3. Results

### 3.1. Pilot Study

The pilot study revealed that participants' comprehension of items and the format of the scale were appropriate in this version of LARS. All participants defined the scale as easy to complete. The mean completion time was 10.1 ± 0.5 minutes. The Kappa coefficient for interrater reliability was 0.93 (95% confidence interval, CI 0.86–0.99; *P* < 0.001).

### 3.2. Experimental Study

The mean age was 71.6 ± 8.1 in PD patients and 69.4 ± 8.7 years in healthy controls. Demographic and clinical characteristics of participants are shown in Table 1. No significant differences were found between the two groups with regard to age, gender, or education level. The median time from disease diagnosis was 49.0 months (range: 26.8–113.2). According to the Hoehn and Yahr scale, 26.2% of PD patients were in stage I, 55.4% in II, 16.2% in III, 1.5% in IV, and 0.8% in stage V. The mean UPDRS part III score (motor examination) in PD patients was 22.9 ± 10.9. Significant differences were found between MEC and MADRS scores from each group (*P* < 0.001). Total LARS scores were different (*P* < 0.001) between PD patients (−14.5 ± 9.1) and healthy controls (−25.0 ± 5.5). The prevalence of apathy in our PD population tested by LARS was 42%. No apathic controls tested by LARS were found in this study.

### 3.3. Psychometric Properties of the Scale

None of the items presented a ceiling effect; however, a floor effect was found for items 20, 21, 24, and 32. The lowest index of discrimination was observed in items 20, 21, 24, 31, 32, and 33. In the study of internal consistency of the scale, the correlation matrix revealed low score correlations between items in different domains and high correlations between items in the same domain. The lowest coefficient was −0.28 (between items 1 and 28), and the highest, 0.45 (between items 15 and 16). Correlations between items in the same domain showed a maximum of 0.45 (between items 15 and 16) and a minimum of −0.10 (between items 30 and 31). LARS Cronbach's *α* was 0.81, with a mean interitem value of 0.11 ± 0.01. However, when items 5, 7, 17, 18, 24, 25, 32, or 33 were removed, *α* value increased. The maximum and minimum correlations between each item and the total score on that item's subscale were 0.90 and 0.31, respectively. The mean value of intersubscale correlation was 0.32 ± 0.03 (highest value = 0.71, and lowest = 0.06). Analysis of the correlation between each subscale score and the total LARS score revealed an *α* value of 0.76 (maximum = 0.71, and minimum = 0.41). The test-restest correlation coefficient was 0.97 (95% CI: 0.94–0.99; *P* < 0.001). Similarly, calculating the correlation using Pearson's analysis yielded a coefficient of 0.97 (*P* < 0.001).

**Figure 1:**
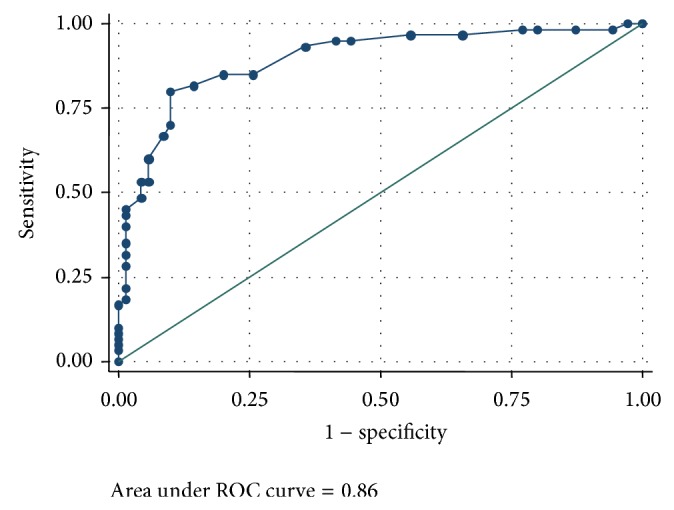
ROC curve for the variable total score for LARS. LARS punctuation versus apathy.

After the PCA, four factors were identified presenting eight values equal or more than 1 which explained 67.5% of the total variance. Factor 1, intellectual curiosity (IC), included the subscales EP, INT, and INI; factor 2, emotion (E), grouped subscales NS, M, and SL; factor 3, action initiation (AI), was composed only by subscale C; and factor 4, self-awareness (SA), included subscales SA and ER. Factor loadings and correlation after oblique rotation are shown in Table 2. The correlation among LARS and NPI scores (concurrent validity assessment) was moderate (*r* = 0.61). Specifically, the maximum and minimum correlations with NPI were found with factors IC (*r* = 0.63) and E (*r* = 0.24) from LARS, respectively. ROC analysis estimated an AUC of 0.86 (95% CI: 0.84–0.95, *P* < 0.001). The cutoff point for an optimal sensitivity and specificity pair was −14 (Table 3). Furthermore, this cutoff presented the maximum Kappa coefficient (*κ* = 0.70). The mean LARS score for PD patients was −14.51 ± 9.15 and for healthy controls, −25.00 ± 5.52 (Figure 1). Calculating patients' cutoff value from the total LARS score with respect to the healthy controls (2.5 SD points below their mean score) yielded a value of −11. So; values higher than −14 are indicative of apathy.

On the other hand, total LARS scores have been shown to depend significantly (*P* = 0.033) on the presence of depression (mean score −9.82 ± 2.14) or nondepression (−14.66 ± 0.67). Similarly, the total score showed dependence (*P* = 0.002) on the presence of cognitive impairment (mean score −10.80) or its absence (−15.36). However, the interactions between apathy diagnosis and depression or cognitive impairment were not significant in either case. Therefore, apathy is independent of both disorders.

Analysis of correlations between the total LARS score and the demographic and clinical characteristics of subjects showed that only age, Hoenh and Yahr stage, UPDRS (parts I, II, and III), MADRS, and MATTIS presented significant correlations (*P* ≤ 0.05). The highest value was found in UPDRS part I (*r* = 0.58) as is shown in Table 4. Patient characteristics included in the multiple regression model were Hoehn and Yahr stage, UPDRS parts I and III, MADRS, MATTIS, and age. These variables explained 34.36% of the variance of the model (adjusted *R* = 0.46; *P* < 0.001, Table 5). Only UPDRS part I, MADRS, and MATTIS presented an independent and significant correlation with total score of LARS which was positive in the case of UPDRS part I (*β* = 0.91, *P* = 0.010) and MADRS (*β* = 0.40, *P* = 0.001) and negative for MATTIS (*β* = −0.19, *P* = 0.002).

## 4. Discussion

LARS is a novel instrument able to identify apathy in patients and differentiate it from depression [11, 23]. To date, only one apathy rating scale has been validated in Spanish and in a cohort of patients from Spain [24]. Data from the present study confirm the efficacy of the Spanish version of LARS for detecting apathy in these patients which provide a new tool to assess patients with Parkinson's disease.

Almost all the participants considered the scale to be very comprehensible and easy to complete. The value of interrater reliability was 0.93, indicating almost perfect agreement. Only four items (21, 24, 32, and 33) presented a floor effect and low discrimination index, probably because these answers may be conditioned by common social-demographic standards. The internal consistency of the scale was high, with a Cronbach's *α* value of 0.81 (appropriate for diagnostic purposes) and half-split reliability of 0.82. The mean interitem correlation was 0.11 ± 0.01. The decrease of *α* observed when most items were removed from the calculation did not result in a value inferior to the lower threshold (0.20). Furthermore, the same decrease observed when removing subscales from the calculation did not result in an *α* value inferior to 0.70. Internal consistency values obtained in this Spanish version were similar to those in the original version of LARS [11]. In general, the scale demonstrates appropriate interitem correlation, few random errors, and high accuracy. As in the original version, the scale presented high interrater and test-retest reliability. The four factors identified after the PCA (IC, E, AI, and SA) were not constituted by the same subscales used in the original version [11]; however, factors from both scales describe the same dimensions of apathy. In the original version, the authors correlated LARS with AES (*r* = 0.87) to study concurrent validity; factors IC and AI presented the strongest values. In the present study, the correlation was performed among LARS and NPI, resulting in a moderate correlation (*r* = 0.61) with good values for IC (*r* = 0.30) and AI (*r* = 0.47). The optimal cutoff point under the ROC curve was −14, whereas the value derived from healthy controls was −11. From these results it may be assumed that some patients who are clinically diagnosed with apathy will not be detected by the scale. For this reason, scores ranging between −14 and −11 should be considered indicative of slight degree of apathy. Therefore, subjects presenting scores below −14 are considered nonapathetic, while scores above −11 indicate moderate/severe apathy. These cutoff values differ from those in the original version of LARS (−17 to −22 cuttoff range) probably due to different characteristics of the participants and their cultural differences. Compared to the original population [11], our PD patients were older; subjects presented lower MATTIS scores, lower educational levels, and longer duration of disease. These features might be characteristic of the Spanish population. Therefore, the cutoff values given in the present study may be recommendable for Spanish subjects. More investigations are needed to corroborate this consideration. Regarding other neurological disorders, the total LARS score was found to be influenced by the presence of depression and cognitive impairment. However, both disorders were shown to be independent identities with respect to apathy.

The limitations of the study are that high educational level patients and PD patients with IV or V of Hoenh and Yahr are underrepresented. Another limitation is to take like gold standard to validate the scale the diagnosis of apathy based on clinical criteria. The literature review does not provide sufficient data sensitivity and specificity of these diagnostic criteria. Apathy is still a controversial entity and difficult to define for many researchers and clinicians. Finally the scale is administered to the patient; therefore anosognosia can be a limiting factor in interpretation of the results. Also in demented patients the difficulty in understanding the items and anosognosia, too, may be biased patient response. The authors of the original scale have developed the caregivers' version of LARS apathy scale [25]. The validation of this scale has also shown very good reliability and validity data. The most important difference is being found useful in demented patients.

The prevalence of apathy in our population assessed by LARS was 42%. This value is in agreement with prevalence data from the literature [1, 2]. Having a Spanish version of LARS that has been validated in subjects from Spain represents a main goal in the management of apathy in Spanish PD patients.

## 5. Conclusion

The Spanish version of LARS is a reliable and useful tool for diagnosing apathy in PD patients. Depression and cognitive impairment influence the total LARS score; however, both entities are independent of apathy. The satisfactory reliability and validity of the scale make it an appropriate instrument for screening and diagnosing apathy in clinical practice or for research purposes.

## Figures and Tables

**Table 1:** Demographic and clinical characteristics of PD patients and healthy controls.

	PD patients (*n* = 130)	Healthy controls (*n* = 70)
Age (years), mean ± SD	71.6 ± 8.1	69.4 ± 8.7
Gender, male/female	78/52	39/31
Education level, *n* (%)		
No studies	15 (11.5)	13 (18.6)
Primary	82 (63.1)	38 (54.3)
Secondary	25 (19.2)	12 (17.1)
High	8 (6.2)	7 (10.0)
Duration of disease, median months (range)	49 (26.8–113.2)	
Hoenh and Yahr scale, *n* (%)		
Stage I	34 (26.2)	
Stage II	72 (55.4)	
Stage III	21 (16.2)	
Stage IV	2 (1.5)	
Stage V	1 (0.8)	
UPDRS score, mean ± SD		
Part I	2.9 ± 2.3	
Part II	7.8 ± 5.7	
Part III	22.9 ± 10.9	
Part IV	1.6 ± 3.2	
MEC score, mean ± SD	30.7 ± 3.8	33.3 ± 1.7∗
MADRS score, mean ± SD	8.6 ± 4.6	2.9 ± 3.7∗
LARS score, mean ± SD	−14.5 ± 9.1	−25.0 ± 5.5∗
MATTIS score, mean ± SD	127 ± 14	
NPI, median ± SD	1.6 ± 2.1	
Semantic fluency score, mean ± SD	14.4 ± 4.8	
Phonemic fluency score, mean ± SD	9.1 ± 4.8	
Clock Drawing Test score, mean ± SD	8.9 ± 2.2	

PD: Parkinson's disease; SD: standard deviation; UPDRS: Unified Parkinson Disease Rating Scale; NPI: neuropsychiatric inventory; LARS: Lille apathy rating scale; MEC: Mini-Mental State Examination; MADRS: Montgomery and Åsberg depression rating scale; MATTIS: Mattis Dementia Rating Scale. ∗Significant differences between both groups, *P* < 0,001.

**Table 2:** Factor loadings after oblique rotation and correlation between oblique factors.

	Factor 1 (IC)	Factor 2 (E)	Factor 3 (AI)	Factor 4 (SA)
Factor loadings after oblique rotation				
EP	0.805	−0.318	0.381	0.033
INT	0.713	0.156	−0.016	−0.049
INI	0.592	0.295	0.087	−0.003
NS	0.249	0.436	0.300	0.077
M	0.214	0.711	−0.214	0.040
SL	−0.185	0.851	0.269	−0.043
C	0.222	0.115	0.810	−0.042
ER	0.223	−0.002	−0.298	0.760
SA	−0.242	0.018	0.322	0.776
Correlations between oblique factors				
IC	1.000	0.381	0.053	0.239
E	0.381	1.000	0.135	0.275
AI	0.053	0.135	1.000	0.158
SA	0.239	0.275	0.158	1.000

EP: everyday productivity; INT: lack of interest; INI: lack of initiative; NS: extinction of novelty seeking; M: motivation; SL: poor social life; C: lack of concern; ER: blunting of emotional responses; SA: extinction of self-awareness; IC: intellectual curiosity; E: emotion, AI: action initiation.

**Table 3:** Sensitivity, specificity, and validity indices and cutoff values for LARS with respect to the clinical diagnostic of apathy performed by the main researcher.

Cutoff point	Accuracy	Kappa	Sensitivity (95% CI)	Specificity (95% CI)
≥−13	0.81	0.61	70.0 (57.6–82.4)	90.0 (82.3–97.7)
≥−14	0.85	0.70	80.0 (69.1–91.0)	90.0 (82.3–97.7)
≥−15	0.83	0.67	81.7 (71.0–92.3)	85.7 (76.8–94.6)

CI: 95% confidence interval.

**Table 4:** Analysis of correlations between the total LARS score and the demographic and clinical characteristics.

	Correlation *r* (*P*)
Age	0,29 (0,001)
Sex	0,076 (0,797)
Educational level	0,025 (0,333)
Time evolution of PD	0,008 (0,924)
Hoenh and Yahr	0,37 (0,000)
UPDRS I	0,58 (0,000)
UPDRS II	0,19 (0,035)
UPDRS III	0,37 (0,000)
UPDRS IV	0,08 (0,382)
MADRS	0,43 (0,000)
MATTIS	−0,47 (0,000)

**Table 5:** Multiple regression model.

Factor∗	*B* coefficient	Bs coefficient∗	Standard error	IC 95%	*P*
UPDRS I	0,91	0,23	0,35	0,21 a 1,60	0,010
MADRS	0,49	0,25	0,15	0,20 a 0,78	0,001
MATTIS	−0,19	−0,29	0,06	−0,31 a −0,07	0,002
Hoehn and Yahr	0,77	0,06	1,20	−1,60 a 3,14	0,519
Age	0,10	0,08	0,08	−0,07 a 0,26	0,242
UPDRS III	0,02	0,23	0,08	−0,15 a 0,18	0,851

^*^Sorted by value *B*. ∗Standardized coefficient *B*.
